# Programmed Death-1 and Its Ligand Are Novel Immunotolerant Molecules Expressed on Leukemic B Cells in Chronic Lymphocytic Leukemia

**DOI:** 10.1371/journal.pone.0035178

**Published:** 2012-04-19

**Authors:** Maciej Grzywnowicz, Joanna Zaleska, Daniel Mertens, Waldemar Tomczak, Paulina Wlasiuk, Kamila Kosior, Agnieszka Piechnik, Agnieszka Bojarska-Junak, Anna Dmoszynska, Krzysztof Giannopoulos

**Affiliations:** 1 Department of Experimental Hematooncology, Medical University of Lublin, Lublin, Poland; 2 Department of Internal Medicine III, University of Ulm, Ulm, Germany; 3 Cooperation Unit “Mechanisms of Leukemogenesis", German Cancer Research Center, Heidelberg, Germany; 4 Department of Hematooncology and Bone Marrow Transplantation Unit, Medical University of Lublin, Lublin, Poland; 5 Department of Clinical Immunology, Medical University of Lublin, Lublin, Poland; Baylor College of Medicine, United States of America

## Abstract

Programmed death-1 (PD-1) is an immunoreceptor predominantly expressed on exhausted T cells, which through an interaction with its ligand (PD-L1), controls peripheral tolerance by limiting effector functions of T lymphocytes. qRT-PCR for PD-1, PD-L1 and their splicing forms as well as flow cytometric assessment of surface expression was performed in a cohort of 58 chronic lymphocytic leukemia (CLL) patients. In functional studies, we assessed the influence of the proliferative response of leukemic B-cells induced by IL-4 and CD40L on PD-1 transcripts and expression on the protein level. The median level of PD-1, but not PD-L1, transcripts in CLL patients was higher in comparison to healthy volunteers (HVs, n = 43, p = 0.0057). We confirmed the presence of PD-1 and PD-L1 on the CLL cell surface, and found the expression of PD-1, but not PD-L1, to be higher among CLL patients in comparison to HVs (47.2% vs. 14.8%, p<0.0001). The Kaplan-Meier curves for the time to progression and overall survival in groups with high and low surface expression of PD-1 and PD-L1 revealed no prognostic value in CLL patients. After stimulation with IL-4 and CD40L, protein expression of PD-1 was significantly increased in samples that responded and up-regulated CD38. PD-1, which is aberrantly expressed both at mRNA and cell surface levels in CLL cells might represent a novel immunotolerant molecule involved in the pathomechanism of the disease, and could provide a novel target for future therapies.

## Introduction

Chronic lymphocytic leukemia (CLL) is the most common adult leukemia in the western population and it is characterized by a heterogeneous clinical course [1]. Mechanisms of CLL pathogenesis are not fully described yet. However, there is growing evidence for the involvement of external microenviromental and internal genetic and epigenetic alternations [1]. Emerging data underlines the key role of the B-cell receptor (BCR) in CLL transformation and progression [1]. Functional BCRs are responsible for antigen-mediated stimulation of both normal and malignant B cells. However, in CLL cells the BCR is weakly expressed [1], [2]. It is noteworthy that several factors involved in BCR signaling have impact on the biology and prognosis of CLL. In the leukemic cells, an aberrant expression of 70 kDa tyrosine kinase zeta-associated protein (ZAP-70), which takes part in the BCR signal transduction pathway, correlates with poor prognosis [3]. Presence of an unmutated gene of the variable regions of the immunoglobulin heavy chain (*IGHV*) appears in about half of CLL patients and is correlated with an unfavorable prognosis [4]. Additionally, over 20% of leukemic cells are carrying stereotyped BCRs, which might suggest a role for antigenic drive in CLL pathogenesis [5]. Stereotyped receptors are more frequent in CLL cell with unmutated *IGHV* genes [6].

Programmed death-1 (PD-1, CD279), a member of the CD28 receptor family, is expressed temporally on T and B lymphocytes upon their activation and binds programmed death ligand-1 (PD-L1, B7-H1, CD274) and PD-L2 (B7-DC, CD273). Interactions of PD-1 with PD-L1 and PD-L2 are well described for T cells, where they inhibit proliferation, cytokine production and cytotoxic capabilities, characterizing thereby “exhausted" T cells [7], [8]. PD-1, attenuates T cells response and thereby plays a role in maintenance of peripheral tolerance [9]. The function of this receptor on tumor cells is unknown. However, up-regulated PD-L1 expression was described in several human tumors types, including hematological malignancies [9], [10], [11], [12]. In T cells, PD-1 inhibits the transduction of T-cell receptor (TCR) signal by blocking ZAP-70 phosphorylation and preventing phosphatidylinositol 3-kinase (PI3K) activation by CD28, which inhibits functions of AKT and extracellular signal-regulated kinase (ERK) [13], [14]. The interaction between PD-L1 and PD-1 leads to deactivation of molecules involved in BCR signal transduction pathway including Syk, PLCγ, ERK1/2, B-cell linker protein (BLNK) and PI3K as well as it is blocking activation of ZAP-70 in T cells [15].

PD-1 is expressed on activated lymphocytes and up-regulated upon their stimulation [16]. Since i) the phenotype of CLL cells has several features characteristic for activated, antigen experienced B cells, ii) PD-1 expression is present in microenvironment of other B-cell malignancies, iii) CLL has some features of T-cells including ZAP-70, CD-5 and CD38, characterization of PD-1 and PD-L1 expression might give deeper insight into CLL biology [17].

## Results

### Differential mRNA expression of PD-1, Δexon2,3,4 PD-1 and Δexon2 PD-L1 splicing variants in CLL patients

For 32 patients samples isolated from PBMCs and cells isolated from BM of 11 patients were analyzed using qRT-PCR. In further analyzes, the tissue source of the analyzed cells showed no significant differences, and therefore in subsequent experiments samples were analyzed collectively.

**Figure 1:**
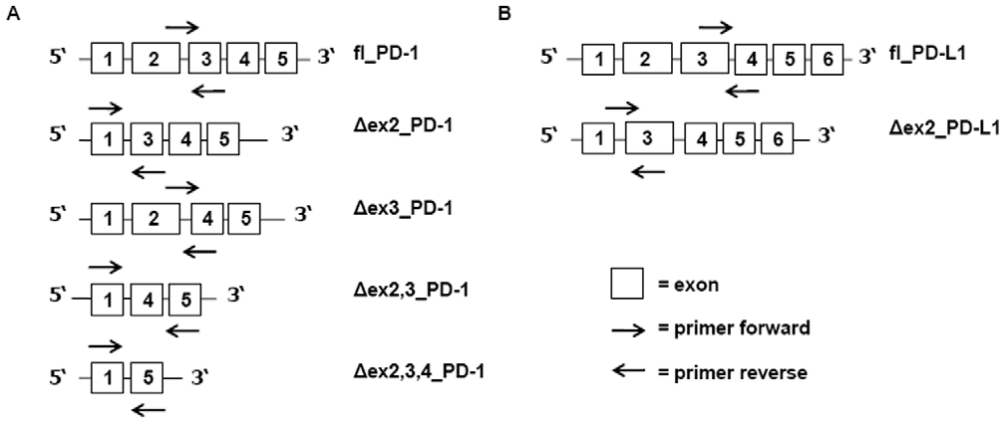
Schematic representation of the organization of PD-1 (A) and PD-L1 (B) splicing variants. Primer localization is marked with arrows. Primers were designed to anneal at exon junctions that are specific for the particular splicing variant.

The organization of PD-1 and PD-L1 splicing variants is presented in Figure 1. The level of full length (fl_PD-1) transcript of PD-1 was elevated in CLL patients in comparison to HVs, with a median relative fl_PD-1/GAPDH expression of 0.57 vs. 0.12, p = 0.0057 (Table 1). The levels of mRNAs splicing variants lacking of exon 2 (Δex2_PD-1), exon 3 (Δex3_PD-1) and both exons (Δex2,3_PD-1) showed no significant differences between HVs and CLL samples. Expression of PD-1 transcripts lacking exons 2, 3 and 4 (Δex2,3,4_PD-1) was higher in HVs than in CLL patients (p = 0.0465). No difference in the expression of the full length PD-L1 (fl_PD-L1) splicing variant was observed between the two analyzed groups. However, the PD-L1 splicing variant lacking exon 2 (Δex2_PD-L1) was highly expressed in CLL patients in comparison to HVs. Exemplary amplification and dissociation curves for PD-1 and PD-L1 slicing variants are displayed in Figure S1.

**Table 1:** Median expression of PD-1 and PD-L1 splicing variants.

Splicing variant	CLL patients	HVs	Statistical significance
fl_PD-1	0.5699	0.117	Yes (p = 0.0057)
Δex2_PD-1	0.1287	0.1208	No
Δex3_PD-1	0.1748	0.135	No
Δex2,3_PD-1	0.1018	0.06951	No
Δex2,3,4_PD-1	0.1027	0.1993	Yes (p = 0.0465)
fl_PD-L1	0.4297	0.2648	No
Δex2_PD-L1	0.3571	−0.2649	Yes (p = 0.0085)

The expression levels of PD-1, PD-L1 and their splicing variants were calculated as an inverse ratio of the difference in cycle threshold (ΔCt) method, where ΔCt is the Ct value of the target splicing variant minus the Ct value of GAPDH.

### Expression of PD-1 and PD-L1 do not correlate with clinical parameters of CLL patients

To further characterize PD-1 and PD-L1 expression, we correlated their expression with clinical parameters of CLL patients. There was no correlation between fl_PD-1 and fl_PD-L1 expression and sex, age, disease stage according to the Binet staging system, cytogenetic abnormalities, expression of ZAP-70 and CD38, percentages of regulatory T lymphocytes and level of lactate dehydrogenase.

We analyzed Kaplan-Meier curves for the time to progression (TTP) and overall survival (OS) in groups with high and low expression levels of PD-1 and PD-L1. Neither PD-1 nor PD-L1 expression level has prognostic value for either TTP or OS.

### Expression PD-1 and PD-L1 on CLL cells

**Figure 2:**
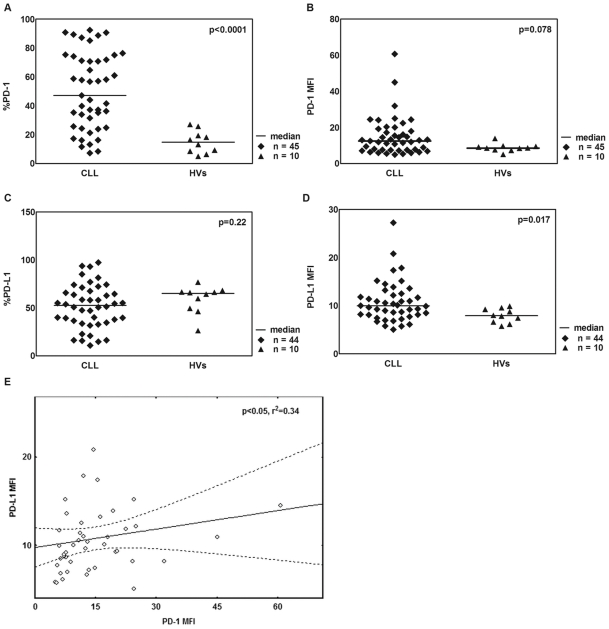
Surface expression of PD-1 and PD-L1 on cells from CLL patients and HVs. Figure displays a flow cytometric analysis of PD-1 and PD-L1 expression on CLL cells and normal B cells. (A) Median PD-1 expression of PD-1 on CD5^+^CD19^+^ CLL cells and control B cells of healthy volunteers (HVs) (47.2% vs. 14.81%, p<0.0001). (B) The mean fluorescence intensity MFI of PD-1 on CD5^+^CD19^+^ CLL cells and control B cells of HVs (12.49 vs. 8.59, respectively, p = 0.078). (C) Median PD-L1 expression on CD5^+^CD19^+^ CLL cells and control B cells of HVs (median: 52.52%, range 10.8%–97.3%, p = 0.22). (D) PD-L1 MFI of CD5^+^CD19^+^ CLL cells and control B cells of HVs (9.96 vs. 7.93, p = 0.017). (E) Correlation of MFI of PD-1 positive CLL cells with PD-L1 positive CLL cells (r^2^ = 0.34, p<0.05). MFI of PD-1 and PD-L1 on CD5^+^CD19^+^ leukemic cells and on CD19^+^ control B cells was defined by flow cytometric analysis (see Figure S2).

To confirm protein expression of PD-1 on CD5^+^CD19^+^ cells isolated from 45 CLL patients, flow cytometry analysis was performed and revealed the presence of PD-1 molecules in all samples. The expression of PD-1 on CD5^+^CD19^+^ was higher as compared to expression on healthy B cells (47.2% vs. 14.81%, p<0.0001, Figure 2A). The representative plot of the flow cytometric analysis of PD-1 expression is presented in Figure S2. Interestingly, the percentage of CD5^−^CD19^+^PD-1^+^ (Figure S2D) was comparable to percentages observed on CD19^+^ B cell of healthy control (Figure S2E). Next, the median of the mean fluorescence intensity (MFI) of the PD-1 molecules on CLL cells and HVs was measured to reflect expression levels. Median MFI of PD-1 tended to be higher on CLL cells than on CD19^+^ cells of healthy individuals (12.49 vs. 8.59, respectively, p = 0.078, Figure 2B)

We also assessed surface expression of PD-L1 on CLL cells from 44 patients and 10 HVs using flow cytometry. Expression of PD-L1 was observed in all samples at similar levels (median: 52.52%, range 10.8%–97.3%, p = 0.22, Figure 2C). The representative plot of the flow cytometric analysis of PD-L1 expression is presented in Figure S2. Interestingly, the MFI values of PD-L1 reflecting the expression levels on CLL cells were higher when compared to HVs (9.96 vs. 7.93, p = 0.017, Figure 2D). The MFI of PD-1 and PD-L1 on CLL cells showed a positive correlation (r^2^ = 0.34, p<0.05, Figure 2E).

### Association of PD-1 and PD-L1 protein expression on CLL cells with clinical parameters of CLL patients

Surface expression PD-1 and its ligand were analyzed with relation to age, sex and stage of disease. No correlation between expression of PD-1 nor PD-L1 and sex, age or a stage of disease was found. There was also no difference of expression of PD-1 between groups of patients characterized by prognostic factors including ZAP-70 and CD38 as well as in different cytogenetic subgroups as defined using the hierarchical model [18]. Interestingly, the expression of PD-L1 was higher in ZAP-70-positive patients (ZAP-70≥20%) as compared to ZAP-70-negative (ZAP-70<20%) cases (58.13% vs. 34.61%, p = 0.0019). We also observed no correlation between PD-1 and PD-L1 expression and the percentages of regulatory T lymphocytes and the level of lactate dehydrogenase.

The Kaplan-Meier curves for the TTP and OS in groups with high and low surface expression of PD-1 and PD-L1 revealed no prognostic value in CLL patients.

### PD-1 expression after stimulation of CLL cells and normal B cells with IL-4 and CD40L

**Figure 3:**
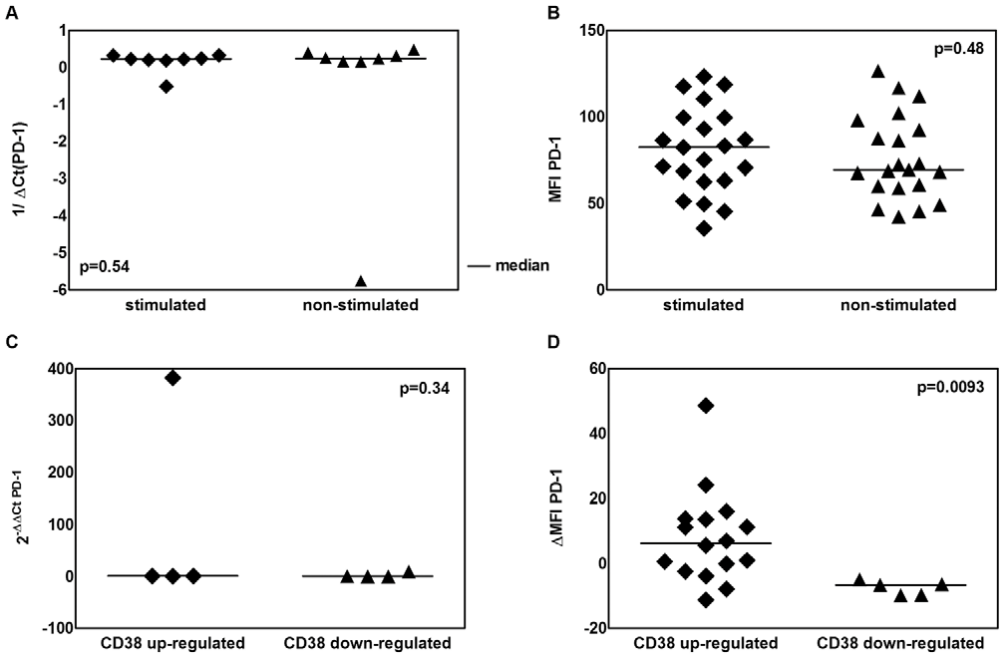
PD-1 expression after CLL cells stimulation with CD40L and IL-4. Figure displays analysis of PD-1 expression on both transcript and protein levels on CLL cells after stimulation with CD40L and IL-4. (A) PD-1 mRNA expression level in stimulated cells and non-stimulated control (0.22 vs. 0.24, p = 0.54). (B) PD-1 MFI in stimulated and non-stimulated cells (82.22 vs. 69.34, p = 0.48). (C) Difference of PD-1 mRNA expression levels between stimulated cells and non-stimulated control assessed using qRT-PCR (2^−ΔΔCt^) and correlated with CD38 MFI, with samples segregated into up-regulation or down-regulation of CD38 upon stimulation (0.637 vs. 0.326; p = 0.34). (D) Change of PD-1 MFI between stimulated and non-stimulated cells, with patients segregated as in (C) (6.240 vs. −6.670, p = 0.0093).

Since our results showed elevated PD-1 expression on CLL cells, in functional studies we characterized its expression after stimulation with IL-4 and CD40L. There were no significant difference between stimulated and non-stimulated CLL cells neither on the PD-1 transcript level (1/ΔCt of 0.22 vs. 0.24, respectively, p = 0.54, Figure 3A) nor surface protein presence (MFI PD-1, 82.22 vs. 69.34, p = 0.48, Figure 3B). In HVs samples, MFI of normal B cells after stimulation was not markedly elevated in comparison to non-stimulated cells (206.5 vs. 212.7, p = 0.59, see Figure S3A)

For further characterization of PD-1 expression after stimulation, we subsequently analyzed CD38 expression, which reflects CLL cells proliferative potential [17]. PD-1 expression was compared in groups of CLL patients and HVs with an up- or down- regulation of CD38 upon stimulation. Levels of CD38 in stimulated cells were compared to non-stimulated control. While there was no differences in PD-1 mRNA levels in CLL cells in both groups (2^−ΔΔCt^ of 0.637 vs. 0.326, p = 0.34, Figure 3C), the ΔMFI of PD-1 was higher in the group who up-regulated CD38 after stimulation (ΔMFI of 6.240 vs. −6.670, p = 0.0093, Figure 3D). The ΔMFI of PD-1 of HVs samples up-regulating CD38 after stimulation was not significantly different than those with down-regulated CD38 (29.17 vs. −36.77, p = 0.33, see Figure S3B).

## Discussion

Programmed death-1 (PD-1) was identified as an inducible molecule on activated T cells, B cells, natural killer T cells, macrophages and dendritic cells [7], [12], [16]. Through an interaction with its ligand (PD-L1), PD-1 controls peripheral tolerance by limiting activation, development and effector functions of T lymphocytes. Expression of PD-1 and its ligand in hematological malignances is still not well described on B cells. Expression of PD-1 was found on tumor infiltrating and peripheral T cells in Hodgkin lymphoma, B-cell non-Hodgkin lymphoma as well as in the adult T-cell leukemia [19], [20], [21], [22]. PD-1 was described also as a marker of T cells associated with germinal centers and as a marker of angioimmunoblastic T-cell lymphoma [19], [23], [24]. Among B-cell non-Hodgkin lymphomas, PD-1 presence was described on 3 of 98 cases of diffuse large B-cell lymphomas (DLBCL), 12 of 13 cases of small lymphocytic lymphoma and 10 of 11 cases of CLL [23], [24]. In our study, we analyzed PD-1 expression on the surface of double positive CD5^+^CD19^+^ cells. We found higher expression of PD-1 both on RNA and protein level in CLL cells when compared to control B cells. Moreover, as a novel feature of CLL cells we observed expression of PD-L1, which has not been described in B-cell non-Hodgkin lymphomas to date [22], [24], [25], [26]. Notably, MFI of PD-L1 was higher on CLL cells than on HVs B-cells, but the level of the full length PD-L1 transcript did not differ between those groups. Correlation of the MFI of CLL cells expressing the receptor and its ligand might indicate simultaneous activation of the signaling pathway of PD-1/PD-L1, which could be a potential mechanism for tumor escape from immunosurveillance.

In order to quantify the expression of PD-1 and PD-L1, we examined alternative isoforms: 5 isoforms of PD-1 and 2 of PD-L1. Δexon2_PD-1 and Δexon2_PD-L1 splicing variants are functionally disabled, since an exon 2 encodes an extracellular IgV-like domain in both cases [27], [28]. We found Δexon2_PD-L1 mRNA to be transcribed with the significantly greater rate in CLL patients than in cells from HVs. Lack of a transmembrane domain encoded by an exon 3 leads to translation of a soluble form of the PD-1 protein (Δexon3_PD-1 transcript). In rheumatoid arthritis, presence of a soluble PD-1 isoform in serum was linked with disease activity characterized by rheumatoid factor in serum and TNF in synovial fluid levels [29]. The Δexon2,3,4_PD-1 transcript has a frame shift caused by alternative splicing that generates a premature stop codon in exon 5 and results in a truncated form of a protein if translated. Interestingly, we found a transcript of Δexon2,3,4_PD-1 to be significantly elevated in cells from HVs in comparison to CLL patients, despite the lack of an obvious biological function of this isoform since exon 4 encodes a cytoplasmatic signaling domain [30].

PD-1 is expressed on T cells and B cells upon their activation, and mRNA levels of all splicing variants are increased in stimulated T cells from HVs [16], [27]. The phenotype of CLL cells reveals multiple features characteristic of antigen-experienced, activated B lymphocytes, that utilize extensively BCR signaling-related molecules including aberrantly expressed ZAP-70 and CD38 [17], [31], [32]. Our results show that surface PD-1 was significantly up-regulated in cells that increased CD38 expression after stimulation, which suggests that PD-1 could be a marker of the activated phenotype of CLL cells. PD-1/PD-L1 is a negative immune system regulator in response to chronic infection and supposedly protects cancer cells. It acts by impairing T-cell functions and their proliferation, and its overexpression on CLL cells upon their stimulation might represent a negative feedback loop in response to stimuli [33], [34]. However, such a regulation *in vivo* appears to be more complex since no general correlation between CD38 and PD-1 expression was found in a larger cohort of CLL patients.

Recently, in follicular lymphoma (FL) the percentage of T cells expressing PD-1 was found to be of prognostic value [35]. However, others showed that PD-1-positive tumor infiltrating T cells are a negative prognostic factor in FL [26]. In the current study, high or low PD-1 expression on either protein or transcript levels has not influence on OS nor on TTP in the group of CLL patients examined here.

Expression of PD-L1 in non-hematological malignances was described in several entities, where it correlated with poor prognosis. Inhibition of the PD-1/PD-L1 pathway was found to be a promising target as it improved the anti-tumor immunological response [7], [36]. In this study we therefore chose to quantify the expression of PD1 in CLL cells. Interestingly, we found higher percentages of PD-L1-positive cells in ZAP-70 positive CLL samples, but analyzes of PD-L1 did not reveal any prognostic value for OS or TTP. In a therapeutic perspective, inhibition of the PD-1/PD-L1 pathway by anti-PD-1 and anti-PD-L1 antibodies has been described as a successful method of improving the anti-tumor response in multiple murine models of human B-cell malignancies, including acute myeloid leukemia [10], [13], [37]. Regarding the reversible “exhaustion" of T cells by effective PD-1 blockade during chronic viral infection [38], a phase I clinical trial for single-dose humanized anti-PD-1 antibody in cancer patients was initiated. Berger *et al.* [39] enrolled 17 patients with different hematological malignancies. In this study, one complete remission in a follicular lymphoma case and stable diseases in both enrolled CLL cases was achieved. Our finding that PD-1 expression is elevated on CLL cells indicates the possibility of using it as a target for immunotherapy.

In summary, we characterized expression of PD-1 and PD-L1 in a cohort of 58 CLL patients. In CLL cells, we demonstrated increased PD-1 expression on both the transcript and the surface protein levels in comparison to healthy donors. By stimulating CLL cells in cell culture with IL-4 and CD40L, we showed PD-1 to be overrepresented in a group of samples that responded to stimulation by up-regulating CD38. We conclude that both PD-1 and PD-L1 might represent novel features of the CLL cell phenotype and might be an interesting target for future therapeutical approaches.

## Materials and Methods

### Ethic Statement

This study was approved by the Ethics Committee of the Medical University of Lublin (No. KE-0254/150/2008). Written informed consent was obtained from all patients with respect to the use of their blood for scientific purposes.

### Patients

Peripheral blood was collected from 58 CLL patients treated in the Department of Hematooncology, Medical University of Lublin, Poland, as well as from 20 age- and sex-matched healthy volunteers (HVs). The clinical characteristics of the patients are summarized in Table 2. For 43 patients assessment of mRNA expression was performed with qRT-PCR. A group of 45 patients was analyzed with flow cytometry, with thirty of them analyzed in both groups.

**Table 2:** Clinical characteristics of CLL patients of groups A and B.

Characteristic	Subcategory	A	B
**Sex**	Female	15	16
	Male	28	29
**Age (years)**	<39	1	1
	40–49	5	5
	50–59	11	8
	60–69	9	12
	70–79	15	15
	>80	2	4
**Binet Stage**	A	17	23
	B	19	15
	C	7	7
**Cytogenetics**	Normal karyotype	8	5
	del13q14	14	15
	del11q22–q23	3	3
	del17p13	3	3
	+12q13	4	3
	Other karyotype	4	3
	Not available	7	13
**ZAP-70 (cut-off 20%)**	Positive	25	27
	Negative	15	13
	Not available	3	5
**CD38 (cut-off 30%)**	Positive	8	9
	Negative	32	31
	Not available	3	5

A – the group of 43 patients analyzed by qRT-PCR for PD-1, PD-L1 and their splicing variants.

B – the group of 45 patients analyzed by flow cytometry method.

Detailed characteristics of CLL patients are presented in Table S1.

### Cell isolation

Mononuclear cells were isolated from peripheral blood (PBMC) or bone marrow (BM) by Ficoll (Biochrom AG, Berlin, Germany) density gradient centrifugation. The viability of obtained cells was always >95% as determined by exclusion of trypan blue. Viable cells were quantified in a Neubauer chamber (Zeiss, Oberkochen, Germany) and stored for further analysis in liquid nitrogen. For total RNA isolation, ten million cells were pelleted and stored in −80°C.

### mRNA preparation and reverse transcription

For total RNA isolation from PBMCs QIAamp RNA Blood Mini Kit (Qiagen, Venlo, Netherlands) was used according to the manufacturer's instructions. From each sample, 1 µg of total RNA was reverse transcribed to 20 µl of cDNA using QuantiTect Reverse Transcription Kit (Qiagen). For quantitative RT-PCR reactions, 1 µl of cDNA of each sample was used.

### Quantitative reverse transcriptase-polymerase chain reaction (qRT-PCR)

For quantitative measurements of the mRNA expression of PD-1, PD-L1 and their splicing variants, quantitative RT-PCR was performed using FastStart Universal SYBR Green Master methodology according to the manufacturer protocol (Roche Diagnostics, Mannheim, Germany). Primers for PD-1 and respective splicing variants (NM-005018) and for PD-L1 and a splicing variant (NM-014143) are described in Table 3. As a constitutively expressed housekeeping gene, glyceraldehyde-3-phosphate dehydrogenase (GAPDH) was used. Thermocycling program was set for 40 cycles of 15 sec at 95°C, 1 min at 60°C with an initial denaturation step at 95°C for 10 min on the ABI Prism 7300 Sequence Detector (Applied Biosystems, Foster City, CA, USA). Particular expression levels were calculated as an inverse ratio of the difference in cycle threshold (ΔCt), where ΔCt is the Ct value of the target splicing variant minus the Ct value of GAPDH.

**Table 3:** Primer sequences.

Transcript name	Primer sequences
fl_PD-1	F: 5′-CTCAGGGTGACAGAGAGAAG-3′ R: 5′-GACACCAACCACCAGGGTTT-3′
Δex2_PD-1	F: 5′-GGTTCTTAGAGAGAAGGGCA-3′ R: 5′-GACACCAACCACCAGGGTTT-3′
Δex3_PD-1	F: 5′-AGGGTGACAGGGACAATAGG-3′ R: 5′-CCATAGTCCACAGAGAACAC-3′
Δex2,3_PD-1	F: 5′-TGGTTCTTAGGGACAATAGG-3′ R: 5′-TCTTCTCTCGCCACTGGAAA-3′
Δex2,3,4_PD-1	F: 5′-TGGTTCTTAGAAGGAGGACC-3′ R: 5′-TCTTCTCTCGCCACTGGAAA-3′
fl_PD-L1	F: 5′-TATGGTGGTGCCGACTACAA-3′ R: 5′- TGCTTGTCCAGATGACTTCG-3′
Δex2_PD-L1	F: 5′-ACGCCCCATACAACAAAATC-3′ R: 5′-CTCTTGGAATTGGTGGTGGT-3′

F, forward; R, reverse; fl_PD-1, full length PD-1; Δex2_PD-1, PD-1 lacking exon 2; Δex3_PD-1; PD-1 lacking exon 3; Δex2,3_PD-1, PD-1 lacking exons 2 and 3; Δex2,3,4_PD-1, PD-1 lacking exons 2, 3 and 4; fl_PD-L1, full length PD-L1; Δex2_PD-L1, PD-L1 lacking exon 2.

### Flow cytometry

For the flow cytometric analysis of the surface expression of the PD-1 and PD-L1, 45 CLL patients (group B, Table 2) were additionally assessed. Initially, CD19 positive cells from PBMC were separated in magnetic field according to the manufacturer's protocol (MACS Milltenyi Biotec, Bergisch Gladbach, Germany). Next CD19 cells were stained with mAbs against surface antigens for 15 minutes at room temperature in the dark. CLL cells were identified in the live cell population using phycoerythrin cyanin 5 (PE-Cy5)-conjugated anti-CD5 mAb (BD Biosciences, Mannheim, Germany). PD-1 and PD-L1 expression was examined using fluorescein isothiocyanate (FITC)-conjugated anti-PD-1 mAb and phycoerythrin (PE)-conjugated anti-PD-L1, respectively (eBiosciences, San Diego, USA). After staining, cells were washed and analyzed by FACSCalibur (BD Biosciences). The results were evaluated by Summit Software (Dako Cytomation, Glostrup, Denmark).

### Flow cytometric analysis of regulatory T lymphocytes

Regulatory T cells were assessed using flow cytometry as CD4^+^ CD25^high^ FOXP3^+^ as described earlier in details [40].

### Leukemic B cell stimulation with IL-4 and CD40L

For functional studies, CD19 positive cells from PBMC cells from 21 CLL patients (median age: 65, sex: 9F, 12M) and from 11 HVs (median age: 58, sex: 6F, 5M) were separated in magnetic field according to the manufacturers protocol (MACS Milltenyi Biotec). Cells were cultured for 24 h in a standard medium consisting RPMI-1640 (Biochrom, Berlin, Germany) supplemented with 10% (v/v) FCS serum, 50 units/ml penicillin, 50 µg/ml streptomycin and 100 µg/ml neomycin with and without addition of 0.01 ng/ml IL-4 and 200 ng/ml CD40L.

After culture, cells were harvested and analyzed for PD-1 expression using qRT-PCR. The difference in PD-1 mRNA expression between stimulated and non-stimulated cells of CLL patients was calculated using the 2^−ΔΔCt^ method, where ΔΔCt represents the difference in ΔCt of PD-1 between stimulated and non-stimulated cells.

Subsequently, after cell culture, flow cytometry analysis was used to assess CD38 expression, which reflects the proliferative response of cells to the stimulation cocktail. In addition, the PD-1 surface expression on CD5^+^CD19^+^ CLL cells was measured for CLL samples and on CD19^+^ normal B cells for HVs samples. The difference in mean fluorescence intensity (MFI) of PD-1 between stimulated and non-stimulated cells was calculated as ΔMFI PD-1.

### Statistical analysis

All results are presented as median values with range. The U Mann–Whitney test was used to evaluate the differences between subgroups of patients and the Wilcoxon signed-rank test for comparing samples in the functional studies. Correlations of variables were computed with the Spearman rank correlation coefficient. The Kaplan–Meier method and the log-rank test were used to assess time to progression (TTP) and overall survival (OS) in different groups of patients.

## Supporting Information

Figure S1 **The amplification (left column) and dissociation curves (right column) for PD-1 and PD-L1 splicing variants by qRT-PCR.**
(TIF)

Figure S2 **Five-parameter flow cytometric analysis of PD-1 and PD-1L expression in chronic lymphocytic leukemia and healthy volunteers.** Figure displays a representative plot of five-parameter flow cytometric analysis for PD-1 and PD-1L after CD19+ magnetic separation (as described in details in methods section). Chronic lymphocytic leukemia (CLL) cells were further gated as CD5 positive cells (A). As compared with control (B) expression of PD-1 on CD5^+^CD19^+^ CLL was measured (C). Other gating strategy on CD5^−^CD19^+^ (D) normal B cells in CLL revealed percentages of PD-1+cells comparable with those observed in healthy controls (E). Simultaneously expression of PD-1L was measured on CLL cells (F).
(TIF)

Figure S3 **PD-1 expression on B cells of healthy controls after stimulation with CD40L and IL-4.** Figure displays (A) PD-1 MFI in stimulated and non-stimulated B cells of healthy volunteers (HVs) (206.5 vs. 212.7, p = 0.59). (B) The difference of PD-1 MFI after stimulation in groups of cases who up-regulated or down-regulated CD38 upon stimulation (29.17 vs. −36.77, p = 0.33).
(TIF)

Table S1 **Clinical characteristics of CLL patients.**
(DOC)
