# Supplementary figures and images for: Programmed Death-1 and Its Ligand Are Novel Immunotolerant Molecules Expressed on Leukemic B Cells in Chronic Lymphocytic Leukemia
Source: PLoS One. 2012 Apr 19;7(4):e35178. doi: 10.1371/journal.pone.0035178 (PMC3331976; doi:10.1371/journal.pone.0035178)

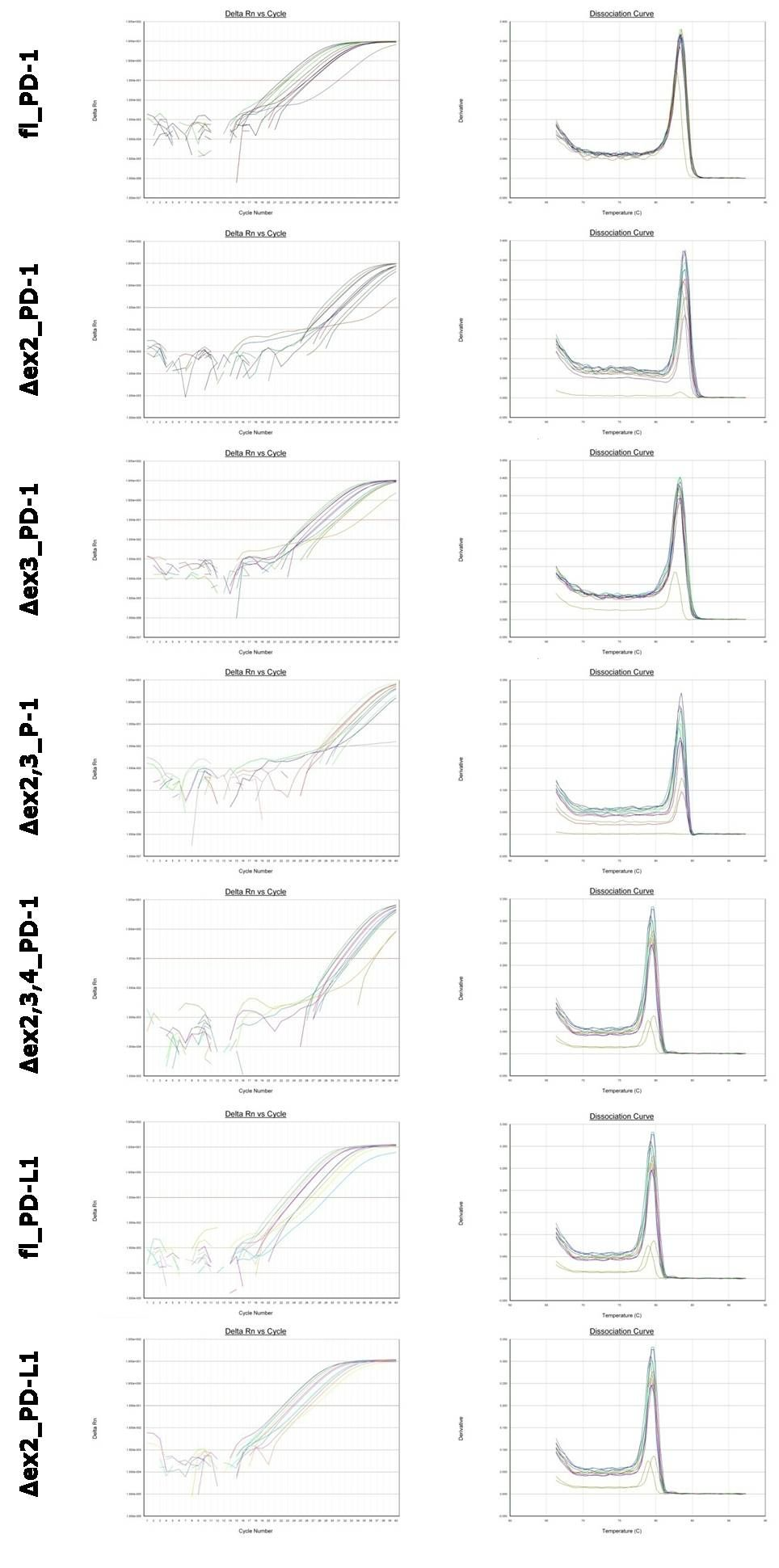

Supplement: Figure S1 — The amplification (left column) and dissociation curves (right column) for PD-1 and PD-L1 splicing variants by qRT-PCR. (TIF) [file pone.0035178.s001.tif]

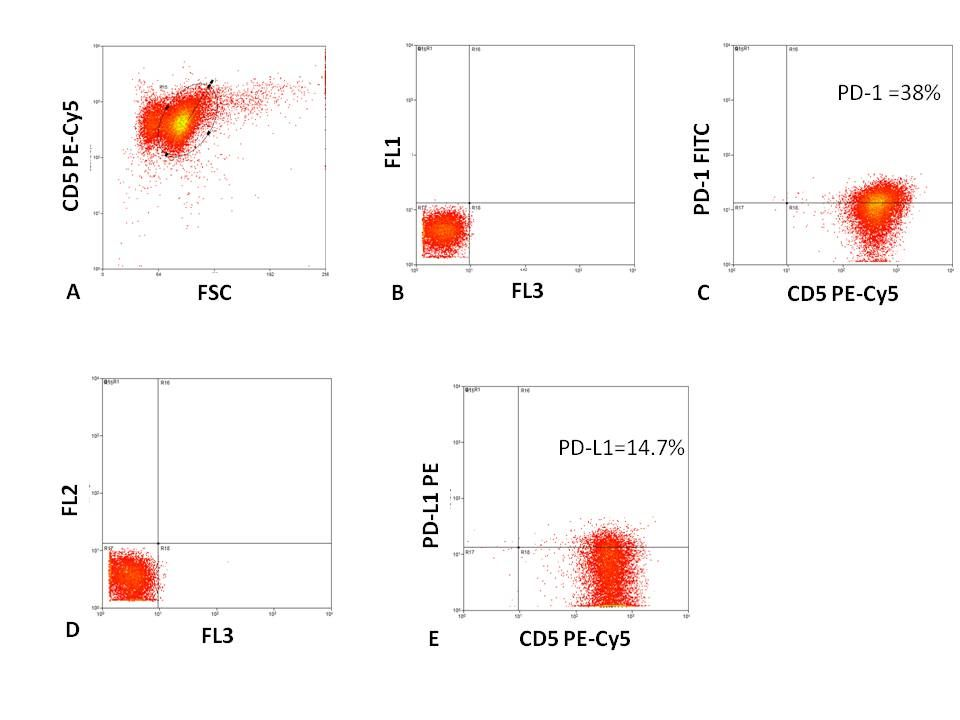

Supplement: Figure S2 — Five-parameter flow cytometric analysis of PD-1 and PD-1L expression in chronic lymphocytic leukemia and healthy volunteers. Figure displays a representative plot of five-parameter flow cytometric analysis for PD-1 and PD-1L after CD19+ magnetic separation (as described in details in methods section). Chronic lymphocytic leukemia (CLL) cells were further gated as CD5 positive cells (A). As compared with control (B) expression of PD-1 on CD5+CD19+ CLL was measured (C). Other gating strategy on CD5−CD19+ (D) normal B cells in CLL revealed percentages of PD-1+cells comparable with those observed in healthy controls (E). Simultaneously expression of PD-1L was measured on CLL cells (F). (TIF) [file pone.0035178.s002.tif]

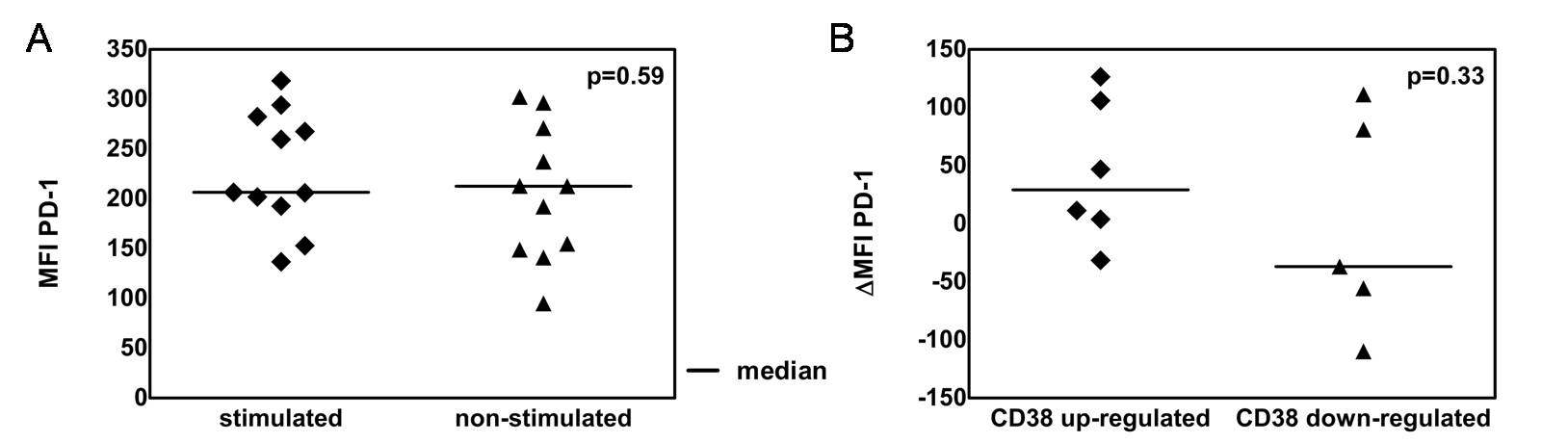

Supplement: Figure S3 — PD-1 expression on B cells of healthy controls after stimulation with CD40L and IL-4. Figure displays (A) PD-1 MFI in stimulated and non-stimulated B cells of healthy volunteers (HVs) (206.5 vs. 212.7, p = 0.59). (B) The difference of PD-1 MFI after stimulation in groups of cases who up-regulated or down-regulated CD38 upon stimulation (29.17 vs. −36.77, p = 0.33). (TIF) [file pone.0035178.s003.tif]
