# Supplementary material for: Programmed Death-1 and Its Ligand Are Novel Immunotolerant Molecules Expressed on Leukemic B Cells in Chronic Lymphocytic Leukemia
Source: PLoS One. 2012 Apr 19;7(4):e35178. doi: 10.1371/journal.pone.0035178 (PMC3331976; doi:10.1371/journal.pone.0035178)
Supplement: Table S1 — Clinical characteristics of CLL patients. (DOC) [file pone.0035178.s004.doc]

|  |  |  |  |  |  | Flow cytometric analysis | | | | | |  |  |
| --- | --- | --- | --- | --- | --- | --- | --- | --- | --- | --- | --- | --- | --- |
| Pts.- no. | Sex | Age | Binet stage | TTP (months) | Chromosomal abnormalities | %PD-1 | MFI PD-1 | %PD-L1 | MFI PD-L1 | Zap-70% | CD38% | 1/ΔCt flPD-1 | Type of sample |
| 1 | M | 76 | A | 22 | normal karyotype | 8.47 | 6.87 | 16.3 | 6.12 | 14.5 | 64.8 | 24.272 | PBMC |
| 2 | M | 61 | B | 10 | del(11), del(13q) | 37.23 | 11.9 | 65.8 | 10.97 | 47.7 | 4.39 | -2.033 | PBMC |
| 3 | F | 71 | A | 4 | n.a. | 47.2 | 13.4 | 97.3 | 27.25 | 36.3 | 26.7 | -1.689 | PBMC |
| 4 | F | 59 | B | 6 | normal karyotype | n.d. | n.d. | n.d. | n.d. | 4.29 | 14.8 | -1.321 | PBMC |
| 5 | F | 54 | A | 15 | del(13q) | 17.3 | 5.52 | 22.7 | 7.74 | 14.4 | 0.8 | -0.330 | PBMC |
| 6 | M | 69 | B | 0.5 | normal karyotype | n.d. | n.d. | n.d. | n.d. | 23.4 | 17 | 0.091 | PBMC |
| 7 | F | 82 | B | 23 | del(13q) | 13.2 | 5.93 | 32.8 | 9.9 | 14.3 | 15.5 | 0.217 | BM |
| 8 | M | 61 | B | 18 | n.a. | n.d. | n.d. | n.d. | n.d. | 44 | 12.5 | 0.227 | BM |
| 9 | M | 59 | A | 50 | del(17p) | 23.9 | 6.29 | 14.8 | 8.5 | 17.72 | 13.08 | 0.227 | BM |
| 10 | M | 66 | A | 19 | trisomy 12 | 71.94 | 15.5 | 74.1 | 17.38 | 28.1 | 71.3 | 0.253 | PBMC |
| 11 | F | 71 | A | 53 | del(13q) | 11.58 | 7.63 | 54.4 | 8.63 | 25.8 | 4.54 | 0.262 | PBMC |
| 12 | M | 78 | A | n.a. | del(11), del(13q) | n.d. | n.d. | n.d. | n.d. | n.a. | n.a. | 0.292 | PBMC |
| 13 | M | 67 | C | 24 | del(13q) | 90.83 | 25 | 40 | 12.18 | 18.2 | 21.3 | 0.313 | PBMC |
| 14 | M | 46 | A | 37 | del(13q) | 41.58 | 11.5 | 85.1 | 12.53 | 22.09 | 0.76 | 0.346 | BM |
| 15 | F | 87 | B | 89 | trisomy 12, del(13q) | 36.27 | 5.98 | 39.4 | 11.7 | 19.4 | 15.4 | 0.363 | PBMC |
| 16 | F | 69 | B | 16 | del(6q) | 44.07 | 17.2 | 64.5 | 10.12 | 23.4 | 67 | 0.375 | PBMC |
| 17 | F | 56 | A | 9 | trisomy 12, del(13q) | 16.11 | 8.06 | 39.7 | 6.97 | 31.3 | 74.5 | 0.404 | PBMC |
| 18 | M | 61 | C | 0.5 | del(17p). trisomy 12 | 24.87 | 9.46 | 55 | 10.03 | 45.3 | 21.8 | 0.408 | BM |
| 19 | F | 72 | A | 13 | del(13q) | 35.36 | 12 | 93.7 | 17.85 | 30.7 | 1.58 | 0.429 | PBMC |
| 20 | M | 76 | B | 27 | del(13q) | 37.22 | 10.8 | 72.4 | 10.58 | 16.89 | 2.86 | 0.461 | PBMC |
| 21 | M | 58 | C | 15 | n.a. | 39.9 | 20.3 | 50.8 | 9.35 | 20.1 | 50.6 | 0.467 | PBMC |
| 22 | F | 73 | A | 13 | n.a. | n.d. | n.d. | n.d. | n.d. | 18 | 4.66 | 0.570 | PBMC |
| 23 | M | 37 | B | 1.5 | del(13q) | n.d. | n.d. | n.d. | n.d. | 12.6 | 3.6 | 0.586 | PBMC |
| 24 | M | 46 | B | 14 | del(6q) | n.d. | n.d. | n.d. | n.d. | 30.9 | 12.5 | 0.604 | PBMC |
| 25 | M | 49 | B | 12 | del(17p), del(13q) | 16.27 | 7.59 | 58.5 | 9.21 | 31.6 | 6.56 | 0.621 | PBMC |
| 26 | F | 55 | A | 1 | normal karyotype | n.d. | n.d. | n.d. | n.d. | 12 | 2 | 0.637 | BM |
| 27 | M | 74 | B | 22 | del(13q) | n.d. | n.d. | n.d. | n.d. | 12.4 | 1.89 | 0.657 | BM |
| 28 | M | 57 | A | 0.5 | normal karyotype | 75.14 | 6.38 | 20.9 | 6.84 | 19.7 | 5.48 | 0.754 | PBMC |
| 29 | F | 60 | C | 0.5 | del(1q), del(13q), tetra(11q), tetra(12q), tetra(13q), tetra(17p), tetra(11;14) | 64.7 | 5.35 | 15.7 | 5.74 | 5.14 | 16.4 | 0.809 | PBMC |
| 30 | M | 79 | A | 14 | del(13q) | 75.41 | 22.4 | 47.1 | 11.84 | 26.8 | 1.72 | 0.832 | PBMC |
| 31 | F | 53 | B | 5 | n.a. | n.d. | n.d. | n.d. | n.d. | 35.7 | 22 | 0.856 | PBMC |
| 32 | M | 75 | C | 10 | trisomy 12 | n.d. | n.d. | n.d. | n.d. | 58.6 | 33.7 | 0.979 | PBMC |
| 33 | F | 48 | A | 5 | del(13q) | 57.88 | 17.9 | 62.9 | 10.9 | 27.3 | 16.9 | 1.045 | PBMC |
| 34 | M | 71 | C | 6 | del(6q), del(11p) | 70.75 | 31.9 | 37.9 | 8.16 | n.a. | n.a. | 1.054 | BM |
| 35 | M | 57 | B | 3 | normal karyotype | 25.65 | 8.88 | 50.6 | 8.1 | 44.1 | 41.9 | 1.084 | PBMC |
| 36 | M | 73 | B | 20 | del(13q) | 35.22 | 7.86 | 53.7 | 13.6 | 17.5 | 49.7 | 1.447 | BM |
| 37 | M | 49 | B | 60 | del(11), del(13q) | 58.82 | 13 | 67.5 | 10.38 | 47.7 | 14.1 | 1.508 | PBMC |
| 38 | M | 67 | B | 0.5 | n.a. | 31.55 | 12.5 | 58.2 | 9.67 | 34 | 1.18 | 2.315 | BM |
| 39 | M | 74 | B | 2 | n.a. | n.d. | n.d. | n.d. | n.d. | 20.8 | 11.7 | 5.928 | PBMC |
| 40 | M | 58 | A | 25 | del(13q) | 90.74 | 24.5 | 81.6 | 15.16 | 69.1 | 4.43 | 5.949 | PBMC |
| 41 | F | 70 | C | 1 | normal karyotype | 70.78 | 16.1 | 77 | 13.23 | 24.6 | 6.86 | 9.497 | PBMC |
| 42 | M | 54 | A | 29 | del(13q) | 92.4 | 7.45 | 47.6 | 15.2 | 41.4 | 2.66 | 20.790 | BM |
| 43 | M | 72 | B | n.a. | normal karyotype | n.d. | n.d. | n.d. | n.d. | n.a. | n.a. | -3.893 | PBMC |
| 44 | M | 75 | A | 28 | n.a. | 7.25 | 7.12 | 55.2 | 8.98 | 21.3 | 1.98 | n.d. | PBMC |
| 45 | F | 76 | C | 0.5 | n.a. | 21.3 | 4.97 | 16.3 | 5.8 | 46.4 | 69 | n.d. | PBMC |
| 46 | M | 36 | A | n.a. | n.a. | 24.38 | 11.2 | 74.3 | 11.42 | n.a. | n.a. | n.d. | PBMC |
| 47 | K | 60 | A | n.a. | n.a. | 34 | 14.5 | 93 | 20.81 | n.a. | n.a. | n.d. | PBMC |
| 48 | M | 81 | A | 31 | n.a. | 56.81 | 13.2 | 33.6 | 7.23 | 23.9 | 3.47 | n.d. | PBMC |
| 49 | K | 67 | A | n.a. | n.a. | 57.13 | 19.2 | 71.3 | 13.88 | n.a. | n.a. | n.d. | PBMC |
| 50 | M | 70 | A | 11 | del(13q) | 58.14 | 14.8 | 36.5 | 7.45 | 18.8 | 0.73 | n.d. | PBMC |
| 51 | M | 74 | B | 14 | del(13q) | 61.03 | 12.9 | 34.6 | 6.7 | 5.8 | 9.54 | n.d. | PBMC |
| 52 | M | 49 | B | n.a. | n.a. | 71.27 | 14.5 | n.d. | n.d. | 17.96 | 0.42 | n.d. | PBMC |
| 53 | M | 95 | A | 0.5 | del(11q) | 74.26 | 7.24 | 31.8 | 8.73 | 45.5 | 3.95 | n.d. | PBMC |
| 54 | M | 79 | B | 6 | n.a. | 76.4 | 24.1 | 40.1 | 8.21 | 24.7 | 15.5 | n.d. | PBMC |
| 55 | M | 64 | B | n.a. | n.a. | 85.2 | 20 | 51.3 | 9.26 | n.a. | n.a. | n.d. | PBMC |
| 56 | M | 74 | A | 0.5 | n.a. | 87.2 | 60.7 | 63.7 | 14.52 | 31.2 | 26.5 | n.d. | PBMC |
| 57 | F | 66 | A | 17 | n.a. | 88.72 | 45.1 | 58.1 | 10.91 | 27.36 | 9.48 | n.d. | PBMC |
| 58 | F | 63 | B | 57 | normal karyotype | 89.4 | 24.4 | 10.8 | 5.06 | 26 | 79.2 | n.d. | PBMC |
